# Mechanical Choices: A Compatibilist Libertarian Response

**DOI:** 10.1007/s11572-023-09671-x

**Published:** 2023-03-31

**Authors:** Christian List

**Affiliations:** Munich Center for Mathematical Philosophy, LMU Munich, Munich, Germany

**Keywords:** Free will, Determinism, Responsibility, Neuroscience, Retributivism, Compatibilism, Libertarianism, Criminal law

## Abstract

Michael S. Moore defends the ideas of free will and responsibility, especially in relation to criminal law, against several challenges from neuroscience. I agree with Moore that morality and the law presuppose a commonsense understanding of humans as rational agents, who make choices and act for reasons, and that to defend moral and legal responsibility, we must show that this commonsense understanding remains viable. Unlike Moore, however, I do not think that classical compatibilism, which is based on a conditional understanding of the ability to do otherwise, provides a sufficiently robust account of free will, even when it is amended as Moore suggests. I argue that free will and responsibility can be defended more robustly by observing that, at the level of agency, there can be alternative possibilities and mental causation in a stronger sense than recognized by classical compatibilism, even if physical determinism is true. Moore’s arguments could thus be strengthened by embracing this compatibilist libertarian position. At the same time, I note that, although the idea of responsibility is robustly defensible, there are independent reasons for rejecting a retributivist approach to punishment.

## Introduction

The significance of neuroscience for the law has recently received much attention. There is a growing chorus of voices arguing that the neuroscientific image of human beings, especially its account of the relationship between the brain and behaviour, dramatically challenges the ideas of free will and responsibility that are central not just to everyday morality but also to the law. The following quotes from the neuroscientist David Eagleman eloquently express the challenge:

“So who is in control? Are you the captain of your own boat, or do your decisions and actions have more to do with massive neural machinery operating out of sight? Does the quality of your everyday life have to do with your good decision making, or instead with dense jungles of neurons and steady hum of innumerable chemical transmissions? … [T]he conscious you is only the smallest part of the activity of your brain. Your actions, your beliefs and your biases are all driven by networks in your brain to which you have no conscious access.”

“There really is no free will; when you arrive at that fork in the road, your choice is predetermined. … [O]ur lives are steered by forces far beyond our capacity for awareness and control.”^1^

One of the lessons, supposedly, is that:

“Criminals should always be treated as incapable of having acted otherwise.”^2^

If this is right, then both morality and the law are mistaken in assuming that people are responsible for their actions. It is our brains and the underlying physical and biological processes that make us do what we do, not our conscious decisions. Neuroscience, so the argument goes, undermines the assumption that people have the sort of control over their actions that is needed for holding them responsible. And therefore, the entire criminal-justice system, with its traditional emphasis on responsibility and its retributivist orientation, stands on shaky ground. If criminals are not responsible for their actions, they do not plausibly deserve to be punished.

In his recent book, *Mechanical Choices*, Michael S. Moore confronts this challenge head-on and offers an ambitious defence of the ideas of free will and responsibility against the neuroscientific criticism, especially in relation to criminal law.^3^ He not only provides a detailed characterization and taxonomy of the relevant neuroscientific arguments but also makes a case for upholding the commonsense understanding of human beings as rational decision-makers who can be held responsible for their actions. As summarized in the book’s abstract, “the book seeks to blunt [the] radical challenges [from neuroscience] while nonetheless detailing how law, morality, and common-sense psychology can harness the insights of an advancing neuroscience to more accurately assign moral blame and legal punishment to the truly deserving”.^4^

My aim in this paper is to offer a constructive critique of Moore’s position. My critique is constructive, insofar as I broadly agree with Moore’s characterization of the neuroscientific challenges and with his claim that, to defend moral and legal responsibility, we must show that the commonsense understanding of humans as rational agents who make choices and act for reasons remains viable. My critique is nonetheless critical, insofar as I do not think that the sort of classical compatibilism that Moore endorses provides a sufficiently robust account of free will. Moreover, regardless of whether a robust account of free will is available, I do not follow Moore in accepting a retributivist approach to punishment.

In short, I make a positive point and a negative one. My positive point is that free will and responsibility can be defended more robustly, by recognizing that, even if physical determinism is true, agents can have alternative possibilities and causal control over their actions in a stronger sense than the conditional one accepted by Moore. Thus I suggest that Moore’s defence of free will and responsibility could be strengthened by embracing this so-called “compatibilist libertarian” position.^5^ My negative point, however, is that, despite the availability of a robust defence of free will and responsibility, there are still good independent reasons for giving up a retributivist approach to punishment and replacing it with an approach that puts more emphasis on restorative justice.

## The Neuroscientific Challenges for Free Will and Responsibility

Moore distinguishes between four challenges that neuroscience poses for free will and moral and legal responsibility.^6^ First, the *challenge from determinism* consists in the claim, allegedly supported by neuroscience’s mechanistic picture of human beings, that everything we do is predetermined by prior physical states, especially physical states of the brain and body. Second, the *challenge from epiphenomenalism* consists in the claim, associated with Benjamin Libet’s famous experiments on the neural causes of voluntary movements, that our conscious choices are not the causes of our actions but that they are mere epiphenomena. The real causes, so it is suggested, are physical events in the brain, such as neuronal readiness potentials, which trigger the conscious experience of choice-making as a byproduct. Third, the *challenge from reductionism* consists in the claim, often made by neuroscientifically inspired philosophers, that we as agents are, at bottom, nothing more than heaps of molecules and neurons, with the implication that the folk-psychological picture of humans as responsible agents is mistaken. Fourth, the *challenge from fallibilism*, allegedly prompted by our growing understanding of the neural underpinnings of human psychology, consists in the claim that we lack the sort of introspective knowledge of, and access to, our own minds that would be needed for genuinely responsible choices. If neuroscience does indeed support all four claims, this speaks against the conventional picture of humans as responsible agents with free will.

I think this taxonomy of the neuroscientific challenges for free will is useful, and I agree with Moore that, to defend the idea of responsibility in the criminal law, we must find some way of answering those challenges. Indeed, my own defence of free will takes as its point of departure a very similar set of challenges, which differs from Moore’s (if we set aside nuances) only in omitting the last challenge.^7^ I also agree with Moore that, even though some earlier versions of those challenges had already been formulated well before the advent of modern neuroscience, the neuroscientific versions of the challenges are particularly serious since they rest on stronger scientific foundations than many of their precursors. For instance, Freudian psychoanalysis might be thought to challenge free will too, but Freudian psychoanalysis is much more controversial from a scientific perspective than modern neuroscience. Finally, there is a fair amount of common ground between Moore and myself with respect to some of the things that might be said in response to the challenges. Yet, I will here critically discuss Moore’s answer to the most widely discussed challenge: the one from determinism.

Moore frames this challenge in terms of a trilemma, which I will restate in slightly modified form.^8^ The trilemma consists in the fact that there appears to be a conflict between three theses that we might expect an account of free will and responsibility to respect:

Physical determinism: Human behaviour takes place against the background of physical determinism, which also underpins the functioning of the brain.

Openness of choices: A necessary condition for free will and moral responsibility is that choices are open, in the sense that their outcomes are not yet settled before those choices are made.

Realism about free will: Free will and moral responsibility are real properties, not just fictions or erroneous ascriptions.

To see the apparent conflict, note that if human behaviour takes places against the background of physical determinism, there appears to be no room for the openness of choices: their outcomes seem predetermined well before those choices are made. But if such openness is necessary for free will and moral responsibility, then free will and moral responsibility cannot be real properties.

It seems to follow that any account of free will and responsibility must reject one of the three theses, and the proponents of any such view must justify why this is acceptable. The disagreement between libertarians, compatibilists, and fictionalists, as Moore presents the debate, lies in which thesis to give up. Let us briefly run through the three responses:

- Libertarians reject the thesis of physical determinism, and they can then uphold the theses that the openness of choices is needed for free will and that, because there truly is such openness, free will and moral responsibility are real properties. The cost of this view is that the rejection of physical determinism may go against what science in general and neuroscience in particular appear (on some interpretations) to teach us about the physical underpinnings of human behaviour.
- Compatibilists, as Moore characterizes them, reject the thesis that the openness of choices is needed for free will and responsibility and replace it with a weaker compatibilist account of what it takes for someone’s choices to count as “free” and “responsible”. The cost of such a view is that it apparently waters down the notions of free will and moral responsibility, possibly below the bar of what is needed to justify our conventional approaches to criminal justice.
- Fictionalists reject the thesis that free will and moral responsibility are real properties and instead hold that they are merely convenient fictions. We may speak *as if* we have free will and moral responsibility, and this way of speaking is useful, but we should not interpret it literally. A moral radical version of this view would be an “error theory”, according to which our talk of free will and moral responsibility is intended to be literally true but rests on a systematic error, just as moral discourse rests on a systematic error according to the error theory in metaethics. The cost of any such view is that it entails that the criminal law is based on a massive fiction or error.

It is worth mentioning that, in Moore’s own exposition of the trilemma, the second thesis differs from the one I have used here. Instead of referring to the “openness of choices”, Moore formulates the thesis in terms of a requirement of “contra-causal freedom”. In his wording, the second thesis asserts that “contra-causal freedom is demanded for moral responsibility”.^9^ The reason I prefer the language of “openness” (instead of “contra-causal freedom”) is that I would like the second thesis to be one that *all* libertarians can accept, and Moore’s wording, unlike mine, seems to exclude some variants of libertarianism.^10^ While all libertarians appear to agree that some form of openness of one’s choices is needed for free will and moral responsibility, not all libertarians will accept that this openness is “contra-causal”. For example, naturalistically inclined libertarians, such as Robert Kane, will presumably want to avoid any violations of the laws of nature or of any causal constraints in their picture of free will, and they will instead look for the sources of openness inside those laws, for instance by relating the relevant openness to quantum indeterminacies in the brain.^11^ Also, agent-causal libertarians will want to say that free choices involve a special form of agent causation, where the act of choice itself initiates a new causal chain, rather than being somehow “contra-causal”.^12^ But even if this could be rendered consistent with what Moore means by “contra-causal freedom”, I think the wording of “openness” is more inclusive.

At any rate, if neuroscience supports the first thesis (physical determinism), our commonsense picture of free agency and responsibility supports the second thesis (openness of choices), and morality and the criminal law presuppose the third thesis (realism about free will), we have a significant problem. What to say in response will be the topic of the next section.

## Moore’s Classical Compatibilism

If, as Moore does, we accept the scientific support for physical determinism and a mechanistic functioning of the brain as independently given (which, for the sake of argument, I won’t question here), and we also follow Moore in considering a fictionalist or error-theoretic account of free will unsatisfactory, especially from the perspective of the criminal law, we must grant the theses of determinism and realism, and it looks as if we must reject the thesis of openness of choices. Indeed, this is the thesis Moore rejects. To be precise, he rejects the version of that thesis formulated in terms of “contra-causal freedom”. Essentially, his account of free will is a classical compatibilist one, inspired by G. E. Moore’s conditional analysis of the ability to do otherwise.^13^

According to such an account, free will does require the ability to do otherwise, that is, an agent’s action counts as free only if the agent could have acted otherwise, but this ability is understood in conditional terms. “The agent could have acted otherwise” is interpreted to mean:

(C) If the agent had tried or wanted to act otherwise, he/she would have succeeded.

As already observed by G. E. Moore, the ability to act otherwise then becomes compatible with determinism. It can be true in a deterministic world that, in the nearest possible world(s) in which the agent had tried or wanted to act otherwise, he or she would have succeeded, which is all that is needed for the truth of (C). In particular, the conditional can be true even if in the actual world the agent was never going to try or want to act otherwise. So, conditional (C) offers a determinism-friendly analysis of the ability to act otherwise. As G. E. Moore wrote,

“[O]ur theory does not assert that any agent ever could have *chosen* any other action than the one he actually performed. It only asserts, that, in the case of all voluntary actions, he *could* have acted differently, *if* he had chosen: not that he could have made the choice.”^14^

Michael Moore recognizes that, despite the initial appeal of this conditional analysis, there are a number of powerful objections suggesting that the analysis does not fully capture the ordinary meaning of ability ascriptions. Most notably, conditional (C) can be true even when there are psychological or physical barriers preventing the agent from trying or wanting to act otherwise. The claim that the agent had the ability to act otherwise seems implausible in such a case. Think of a drug addict whose addiction is so powerful that trying *not* to give in to his addiction is simply too hard. It may be true that *if* this agent tried to act otherwise, he would succeed, but the “if”-clause is too big an “if”. Many compatibilists have therefore chosen to abandon the conditional analysis in favour of a dispositional analysis, according to which to say that an agent could have acted otherwise is to say that:

(D) The agent has a (suitably defined) disposition to act otherwise in (suitably defined) relevant circumstances.^15^

Michael Moore, however, does not find the arguments for a dispositional analysis convincing and instead upholds the conditional analysis, albeit with some amendments. Specifically, he proposes to replace conditional (C) with a pair of conditionals^16^:

(C_action_) If the agent had chosen to act otherwise, he/she would have acted otherwise.

(C_choice_) If the agent had wanted to do so badly enough, he/she would have chosen to act otherwise.

The first of these conditionals is intended to capture the ability to *act* otherwise; the second is intended to capture the ability to *choose* otherwise. For Moore, the *conjunction* of the two conditionals is required for free will. This is meant to address the sort of objection mentioned earlier, namely that, if we focus on only a single conditional such as (C), an agent could count as free even when there are psychological or physical barriers preventing him or her from choosing to act otherwise. In such a case, Moore suggests, the second conditional, namely (C_choice_), is false, even if the first conditional, namely (C_action_), is true, and so the amended analysis can avoid the objection.

Similarly, the use of the two conditionals gives Moore some resources to avoid the tendency of the classical conditional analysis to over-ascribe abilities where there are none. As noted, the classical analysis ascribes the ability to do otherwise even to a drug addict acting under to the constraints of his or her addiction or someone suffering from severe psychological compulsion, even though it is hard or impossible for the person to choose to act otherwise. Ordinarily, we think that the person did not act out of their own free will and had a valid excuse for doing what they did. Moore’s idea, presumably, is that the second conditional is false in such a case: it is not true that if the agent had wanted to do so badly enough, he/she would have chosen to act otherwise. If this is right, Moore’s amended analysis avoids the over-ascription of an ability to do otherwise to the person in question.

Moore’s analysis departs from the classical conditional analysis of abilities in a further respect. He takes the relevant pair of conditionals to be merely a *test* for the ability to act otherwise, not as the *defining condition*. This is meant to address the worry that, in so-called “finkish” worlds, where abilities disappear whenever we are trying to test for them, the conditionals might not correctly express the truth-conditions for ability statements. And so, the conditionals are inadequate as general defining conditions for the ability to act otherwise. Since “finkish” worlds are very contrived and apparently only relevant to philosophical thought experiments, however, I will here concede that having a test for abilities that is accurate in non-finkish worlds is good enough.

Moore also considers a version of the two conditionals in which the consequent clause is formulated using the word “could” instead of “would”, i.e.,

- “One could have acted otherwise” means “if one had chosen to act otherwise, one could have acted otherwise”, and
- “One could have chosen to act otherwise” means “if one had wanted to do so badly enough, one could have chosen to act otherwise”.

However, I consider this version of the conditionals a non-starter because it generates a regress. If the conditional formulation is meant to be a substitute for the original “could” statement, then it remains unclear how we should interpret the word “could” inside those conditionals, where the original “could” statement reappears as the consequent. Therefore, I will set this version of the conditionals aside and will continue to treat (C_action_) and (C_choice_) with the word “would” rather than “could” as the correct version of Moore’s conditionals.

## Why Moore’s Compatibilism is Insufficient for Moore’s Purposes

For the sake of argument, I will concede that Moore’s pair of conditionals (C_action_) and (C_choice_) constitutes an improvement over the classical conditional analysis of the ability to do otherwise, in particular when Moore’s proposal is understood as a test for the ability to choose and act to do otherwise, where the test is intended for non-finkish worlds.

Nonetheless, I will now argue that Moore’s version of the conditional analysis falls short as a condition for free will and responsibility, even if we focus just on the “could have done otherwise” requirement for responsibility while setting aside other requirements, such as intentionality or mental causation. This is especially so when the goal, as in Moore’s project, is to ground what is sometimes called “basic desert moral responsibility”, “the sense [of responsibility] that would make us *truly deserving* of praise and blame, punishment and reward”, to use Gregg Caruso’s words.^17^

The reason, quite simply, is that, on the commonsense understanding of human agency on which ideas of criminal justice are traditionally based, a necessary condition for an agent to count as responsible for something he or she did is that it wasn’t impossible for this agent to do otherwise. If it was impossible for the agent to do otherwise, the agent cannot plausibly be blamed, let alone punished, for doing what he or she did.

This impossibility, however, is far from excluded by the conditionals (C_action_) and (C_choice_) but remains entirely consistent with them. That is, the conditionals (C_action_) and (C_choice_) can come out as true even when doing otherwise would have been strictly impossible for the agent. In such a case, the truth of (C_action_) and (C_choice_) doesn’t license the kind of responsibility ascription that a proponent of a retributivist approach to punishment such as Moore would need to rely on.

To see that (C_action_) and (C_choice_) are jointly consistent with the impossibility of doing otherwise, note that, on the standard semantics for conditionals (familiar, for instance, from David Lewis’s and Robert Stalnaker’s works), a conditional such as “If P were the case, then Q would be the case” is true in the actual world if and only if its consequent Q is true in all nearest possible worlds in which its antecedent P is true.^18^ Thus, for (C_action_) and (C_choice_) to be true in the actual world, it must be the case that:

- In the nearest possible worlds in which the agent chose to act otherwise, he/she did in fact act otherwise; and
- In the nearest possible worlds in which the agent wanted to do so badly enough, he/she did in fact choose to act otherwise.

Crucially, both of these statements can be true even if the “nearest possible worlds” in question are intuitively very remote. For instance, under the assumption of determinism, the nearest possible worlds in which the agent wanted to choose to act otherwise could be ones in which the initial conditions of the universe—say, at the time of the Big Bang—were different from what they actually were, or they could be ones in which the laws of nature were breached at some earlier point in time, so as to put the agent on a different trajectory from the one on which he or she was actually embarked. In particular, it is not a necessary condition for the truth of either of the bulleted statements that the nearest possible worlds in question were accessible from the actual world at some time in the past. And so, Moore’s two conditionals could come out as true even if the scenarios described by their antecedents were historically inaccessible in the actual world. To insist that we should nonetheless say that the agent could have acted otherwise is at best counterintuitive, at worst simply mistaken.^19^

Might one claim that the two conditionals couldn’t both be true if choosing to act otherwise or trying to make that choice had been genuinely impossible? In particular, might it help to argue that, in such case, there would not be any nearest possible world in which the agent chose to act otherwise or one in which the agent wanted to do so badly enough? Unfortunately, this response wouldn’t help at all. If there were no nearest possible worlds in which the agent chose to act otherwise or wanted to make that choice, the two bulleted statements would be vacuously true: if there are no possible worlds at all in which P is true, then any consequent clause Q is vacuously true in any such world.

The bottom line is that (C_action_) and (C_choice_) can both be true even if, in the actual world,

- It was impossible for the agent to act otherwise,
- It was impossible for the agent to choose to act otherwise,
- It was impossible for the agent to want to choose to act otherwise.

It then seems implausible to claim that the agent could bear moral and legal responsibility for doing what he or she did, especially in the sense of “basic desert moral responsibility”. I conclude, consistently with what responsibility sceptics such as Gregg Caruso and Derk Pereboom have argued more generally, that the sort of classical compatibilism that Moore endorses is too weak as a basis for the retributivist understanding of responsibility that Moore wishes to uphold.^20^

## A More Robust Defence of Free Will and Responsibility

The foregoing considerations suggest that, if we wish to defend free will in a sense that vindicates our ordinary practices of assigning moral and legal responsibility, we must overcome the limitations of classical compatibilism. If we accept that an agent can be responsible *only* in cases in which it was *possible* for him or her to act otherwise, then defending the ability to do otherwise in a merely conditional sense isn’t enough. As Susan Hurley noted:

“[T]he ability to do otherwise entails the *outright possibility* of acting otherwise: it entails that there is a causal possibility of acting otherwise, holding all else constant. A counterfactually conditioned disposition to act otherwise is not the same thing as an outright possibility of acting otherwise.”^21^

If we go back to the trilemma discussed above, it seems that we cannot defend the ability to do otherwise in the required robust sense unless we retain the thesis of the openness of choices, which, as readers will recall, is the following:

Openness of choices: A necessary condition for free will and moral responsibility is that choices are open, in the sense that their outcomes are not yet settled before those choices are made.

But if we consider realism about free will and moral responsibility non-negotiable, it seems that we cannot retain this openness thesis in the face of the scientific support for physical determinism. This was precisely the trilemma we began with.

I will now explain, however, that, contrary to first appearances, the theses of physical determinism, openness of choices, and realism about free will are mutually consistent after all: free will in a relatively robust sense does in fact fit into the worldview given to us by the sciences in general and neuroscience in particular. Specifically, I will suggest that science supports the claim that humans have free will in a recognizably libertarian sense, albeit one that—surprisingly—turns out to be compatible with physical determinism. Free will, in the sense to be defended, involves three things^22^:

Intentional agency: Any bearer of free will is an intentional agent: an entity with beliefs, desires, and intentions who interacts with his or her environment in a more or less intelligible, goal-directed manner.

Alternative possibilities: Any bearer of free will faces choices between different courses of action, each of which constitutes a possibility for this agent.

Mental causation: Any bearer of free will has causal control over his or her actions, in the sense that what the agent does is caused by his or her relevant intentional mental states, not merely by some sub-intentional states of the brain and body.

For the purposes of my argument, I will take these three conditions to be jointly necessary and sufficient for free will. Their wording could of course be fine-tuned, but for now I will treat the conditions as sufficiently intelligible, and their meaning—especially that of alternative possibilities—will become clearer in what follows.

The first thing I want to note is that the neuroscientific challenges for free will are based on a fairly reductionistic view of human beings and their behaviour. Those challenges depict human beings essentially as biophysical systems, in which physical, chemical, and biological processes in the brain and body take centre stage. The challenge from determinism consists in the fact that, from such a low-level perspective, human organisms may be governed by deterministic physical laws such that, conditional on the precise physical microstate at any point in time, there is only one possible future series of physical states, which seems to preclude the existence of alternative possibilities. Similarly, the challenges from epiphenomenalism and reductionism consist in the fact that, from a low-level perspective, there appears to be no room for mental causation or even for genuine intentional agency itself. Rather, there appear to be just physical and chemical processes in the brain and body, which neuroscience increasingly helps us to understand. If this is the lens through which we look at human beings and their behaviour, then it is no surprise that we find no support for free will and free action, or even for intentional agency itself.

But despite the insights that neuroscience has given us, its reductionistic approach is appropriate only for *some* explanatory purposes, such as when we seek to explain the low-level neural implementation mechanisms of human cognitive capacities. For other purposes, such as when we want to explain what humans can and cannot do and why they choose to do one thing rather than another, a non-reductionistic, higher-level approach is needed. What I want to argue is this^23^:

**Claim 1**: In many domains of the human and social sciences, it is explanatorily indispensable to depict humans as choice-making agents with free will, i.e., to postulate that they are intentional agents, with alternative possibilities to choose from, and mental causation.

And further:

**Claim 2**: This postulate is consistent with the rest of science.

Suppose, in addition, we rely on the following familiar principle from the philosophy of science^24^:

**The naturalistic ontological attitude**: Whenever postulating some property or entity is explanatorily indispensable in some domain and this postulate is consistent with the rest of science, we are warranted in taking a realist attitude towards that postulate. (Here, the “warrant” is the ordinary scientific one, akin to the warrant we have for believing that electrons, gravity, and electromagnetism are real.)

Then we can conclude this:

**Conclusion**: We are warranted in taking a realist attitude towards the depiction of humans as choice-making agents with free will.

I now want to explain why we should accept the key premises of this argument, namely Claims 1 and 2. That is, I want to explain why ascribing intentional agency, alternative possibilities, and mental causation to people is indispensable for many explanatory purposes, and why this ascription does not conflict with the rest of science. Given space constraints, I will focus on intentional agency and alternative possibilities and omit mental causation. Further details, as well as a discussion of mental causation, can be found in my recent book.^25^

Let me begin with the explanatory indispensability of postulating intentional agency. Think about how the sciences of human behaviour explain phenomena ranging from market transactions and voting behaviour to cultural rituals and everyday social interactions. If we sought to explain those phenomena by viewing people as nothing but biophysical systems—heaps of interacting cells—we wouldn’t know where to begin. As noted, neuroscience might shed some light on the physiological mechanisms by which higher-level cognitive capacities are implemented in the brain. However, without identifying the meaningful, intentional nature of the activities involved in the mentioned phenomena, we have no hope of giving an adequate account of them. The sciences of human behaviour recognize this and explain all the given phenomena by depicting people as intentional agents, with beliefs and desires, goals and plans, on the basis of which they choose their actions.

Of course, disciplines ranging from anthropology and psychology to economics and sociology differ significantly in their methods and approaches, and scholars in those disciplines disagree about the precise nature of intentional agency. For instance, they disagree about how much rationality we should ascribe to intentional agents and how we should conceptualize “rationality”. Nevertheless, the general presupposition that human beings are intentional agents is central to all the disciplines in question, for good scientific reasons. As Daniel Dennett already noted in his classic work on the “intentional stance”, viewing people as mere physical organisms would be unilluminating for many purposes, and viewing them as intentional agents is essential.^26^ So far, this is a widely accepted point.

Now, once we recognize the explanatory indispensability of postulating intentional agency, it also becomes evident that this postulate must be accompanied by a postulate of alternative possibilities. To see this, consider some illustrative questions addressed by intentional explanations in the human and social sciences. Why does someone who has made an appointment usually show up? Why does someone vote for one political party rather than another? Why do consumers respond to price changes? Why do people at least sometimes behave morally? Whenever we answer such questions, I suggest, we either explicitly or implicitly rely on the following three-part hypothesis.^27^

**Explanatory hypothesis**: The agent

1. Has some options, which are his or her possible choices,
2. Deliberates about, or at least cognitively processes, those options (which can take any form ranging from slow and rational to fast and spontaneous), and then
3. Chooses one option among the possible ones, in a way that is more or less supported by the agent’s mental state.

For instance, we cannot give a good explanation of why a particular person is showing up for an appointment without attributing to that person the choice between showing up and not showing up and identifying the reasons as to why the person is making the choice she does. Similarly, we cannot explain why a person is casting his vote for a populist party without attributing to that person a choice in the first place. Without the assumption that the person is making a choice, we would not be able to explain why he ends up choosing one thing over another. The same is true for the other illustrative questions, concerning responses to price changes and moral behaviour. In all these cases, the explanatory strategy involves pointing to the fact that the relevant agent chooses one option rather than another and that this choice is somehow supported by the agent’s mental state.

The key presupposition is that any agent in question sometimes has alternative possibilities to choose from. Or in other words:

Key presupposition: At any given choice node, the agent’s trajectory admits different “agentially possible” continuations, one such continuation for each option that the agent could possibly choose.

**Fig. 1:**
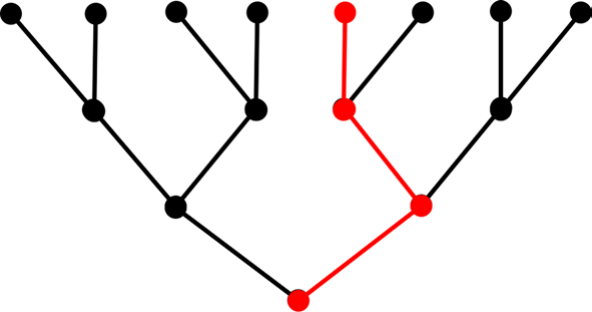
A decision tree

I call this thesis “agential indeterminism”. The thesis suggests that, as people go about their activities, they take some path through an extensive-form decision tree, as illustrated in Fig. 1.

The dots represent choice nodes, and the branches, moving upwards from the root of the tree, represent possible trajectories the agent may take. Even if a given theory of human behaviour singles out one of these trajectories as the predicted one, which the theory says the agent will rationally take (say, the highlighted one), all the other trajectories are deemed possible too. In this sense, any such explanatory theory presupposes what I have called “agential indeterminism”: the availability of alternative possibilities for the agent. Thus, our theories of human behaviour are, in effect, committed to the thesis of the openness of choices that libertarians defend and Moore gives up.

However, even if I am right that postulating intentional agency and alternative possibilities (as well as mental causation) is explanatorily indispensable in the human and social sciences, readers might still worry that this is insufficient to justify a realist attitude towards those properties—and by implication, towards free will—because the relevant postulates might still conflict with the rest of our scientific worldview. In particular, the postulate of alternative possibilities may be at odds with what neuroscience has taught us about the physical determinism underpinning the functioning of the brain. For this reason, that postulate can, at best, be an explanatorily useful fiction. In the next section, I will respond to this worry.

## Why Agential Indeterminism is Compatible with Physical Determinism

I want to explain why physical determinism does not rule out agential indeterminism.^28^ At first sight, one might think that if a system behaves deterministically at some underlying level, say, the level of physical processes in the brain and body, then the system’s behaviour must be deterministic across the board, irrespective of whether we are focusing on the system’s low-level or high-level properties. But this initially plausible thought can be shown to be mistaken.

First of all, we must note that many systems admit more than one level of description. At different levels, we use different concepts and categories to speak about such a system’s behaviour, and we represent the system’s states in a more or less fine-grained manner. Think of the Earth’s atmosphere, for example. We can think of it as a microphysical system in which there are gazillions of air molecules each of which behaves in a deterministic way according to the laws of classical physics. Or we can think of it as a macroscopic system in which certain macro-variables stand in probabilistic relations to one another: a higher temperature, for instance, raises the probability of a thunderstorm. For different explanatory purposes, different descriptions may be more or less appropriate, and they draw our attention to different properties of the system: low-level properties in one case and high-level properties in another. It’s not the case that low-level properties are real and high-level properties fictional, but they are each real, albeit level-specific.

The crucial point, now, is that a system’s macro-behaviour may be indeterministic even when its micro-behaviour is deterministic, so that the determinism-indeterminism distinction is best viewed as a level-relative distinction, rather than a level-independent one. For a proof of concept, I will briefly sketch a toy model. I have developed this model more fully in other work.^29^

**Fig. 2:**
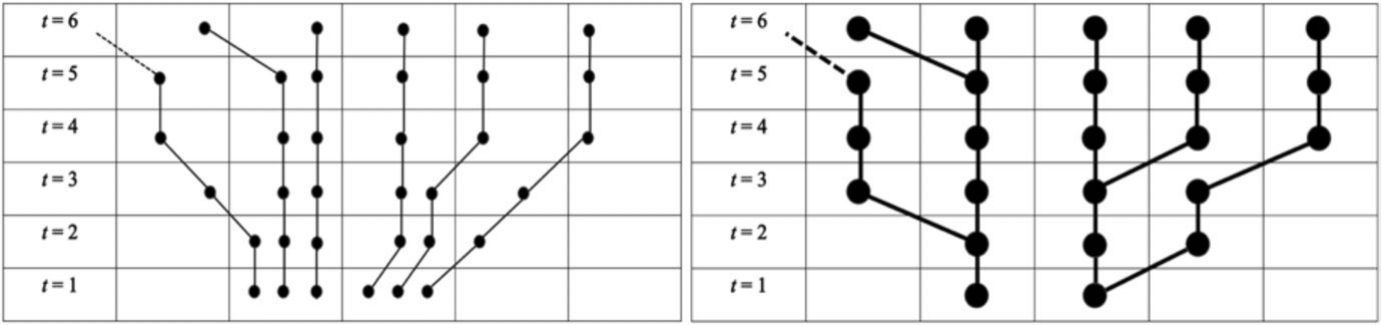
Micro- and macro-level histories

Consider a system whose state evolves over time. At each point in time, the system is in a particular state, which we may interpret as a micro-level state, and some state evolutions are permitted by the laws governing the system, while others are not. A possible state evolution is called a *possible history* of the system. For our example, suppose that all the possible micro-level histories are as shown on the left-hand side of Fig. 2.^30^ Little dots represent states in which the system could be at any point in time (running from time *t* = 1 to time *t* = 6), and lines from bottom to top represent histories. In a fairly clear diagrammatic sense, those histories are deterministic. Given the initial state of any such micro-level history, there is only one possible way the history could evolve over time.

Suppose, however, we are interested not in the system’s micro-level states and histories, but in its macro-states and macro-histories, as describable by some higher-level concepts and categories. In the realm of human behaviour, the macro-level could be that of psychology or the social sciences, as opposed to the level of brain physiology. In accordance with a standard assumption about the relationship between a system’s states at different levels, let us assume that macro-states supervene on micro-states but are multiply realizable by them, i.e., each macro-state corresponds to an entire equivalence class of micro-states, consisting of its possible micro-level realizers. For concreteness, suppose that whenever two or more distinct micro-level states fall within the same cell in the rectangular grid on the left-hand side of Fig. 2, they each realize the same supervenient macro-state. The right-hand side of Fig. 2 shows the resulting macro-level states and macro-level histories, represented by thick dots and thick lines, respectively.

We can see, again in a clear diagrammatic sense, that those macro-level histories are indeterministic. Given the same initial macro-state, the macro-level history could evolve in two or more distinct ways. The initial macro-state is insufficient to determine all subsequent macro-level states.

This simple example shows that indeterminism in macro-level histories can go along with determinism in the underlying micro-level histories. More generally, a system’s dynamics at different levels need not “mesh”, as the philosopher of physics Jeremy Butterfield puts it.^31^ Lower-level determinism can co-exist with higher-level indeterminism, and even the converse is possible too. We may conclude that the sort of physical determinism that is supported by some parts of science, including possibly some parts of neuroscience (if the hypothesis of the mechanical brain is true), does not in any way rule out the sort of agential indeterminism that is presupposed by some of our best explanations in the human and social sciences.

Some critics may object that the kind of “emergent” macro-level indeterminism illustrated by Fig. 2 shouldn’t be viewed as a real phenomenon. Rather, it should be viewed as merely epistemic: it is relative to the informational limitations of our macro-level descriptions that we are unable to predict the macro-history based on information about the initial macro-level state. But this, so the objection goes, does not establish that the system is genuinely indeterministic.

However, since the distinction between determinism and indeterminism is level-relative rather than level-independent, it would be ad hoc to single out some lower level, say, the level of the brain, as the “correct” level at which questions about determinism and indeterminism are to be adjudicated and to dismiss the same distinction at other levels (especially higher ones) as being of no significance. It is much less arbitrary and more systematic to treat the phenomena at *all* levels as genuine aspects of reality. Just as we wouldn’t regard DNA, ecosystems, and institutions as less real than electrons, neutrons, and protons just because they are higher-level phenomena, so we shouldn’t regard higher-level indeterminism as unreal.

More generally, I’d like to note that any definition of determinism is based on some modal notion: a notion of possibility. While the definition of physical determinism is based on the notion of physical possibility, the definition of agential (in)determinism is based on the notion of agential possibility. It is widely accepted that different modal notions have their own legitimate place in science: in addition to the notion of physical possibility, there are also notions of chemical, biological, psychological, and even socio-economic possibility. Agential possibility is the modal notion associated with our best theories of intentional agency. A scientist who is committed to the naturalistic ontological attitude mentioned above has every reason to take a realist attitude towards any modal notion that is well-supported by some corresponding branch of science. And therefore, I conclude that we should be realists about agential possibility and the associated notion of agential indeterminism as much as we are prepared to be realists about physical possibility and whatever the physical sciences say about the determinism-indeterminism distinction at the levels they are concerned with.

What we have arrived at is, in effect, a compatibilist version of free-will libertarianism: an account of free will that can simultaneously respect physical determinism, the openness of choices, and realism about free will. The lesson is that Moore’s trilemma, at least when reframed in the way I have done, is only an apparent trilemma. We don’t need to give up any of the three theses. Interestingly, Moore mentions the idea that humans admit different levels of explanation, citing the work of William Bechtel and Carl Craver, as well as the idea, which he calls the “two-languages view”, that humans may be free at the level of their mental states but determined at the level of the underlying physical brain states.^32^ However, he doesn’t seem to recognize the potential that these ideas offer for a robust defence of free will that retains at least some of the intuitive force of libertarianism. He characterizes the two-languages view, for instance, as a kind of “linguistic fictionalism”.^33^ Instead of embracing the levelled picture, as noted above, he opts for what I would consider a more watered-down account of free will, in the classical compatibilist tradition. But fortunately, as we have seen, a more robust defence of free will can be given.

## Retributivism Revisited

While I have agreed with responsibility sceptics such as Gregg Caruso and Derk Pereboom that a classical compatibilist account of free will is too weak as a basis for a retributivist approach to punishment, this still leaves room for saying that my own compatibilist libertarian account is sufficiently robust to ground responsibility in the required “basic desert” sense. Indeed, that’s the conclusion that a retributivist such as Moore would presumably wish to draw. If Moore considers his own compatibilist defence of free will sufficient for retributivism, then *a fortiori* he should consider the present more libertarian defence sufficient too. However, I now want to turn to the negative point I announced. Although a robust defence of free will is available, I think there are independent reasons for giving up retributivism and replacing it with an approach to punishment that puts the emphasis on restoration and rehabilitation. It would be unfortunate if critics of retributivism felt the need to argue against free will in order to reject retributivism and to advocate criminal-justice reform.^34^ One can be both a staunch realist about free will and a staunch critic of retributivism and of harsh forms of punishment.

Retributivism, as Alec Walen characterizes it, is the view:

1. “That those who commit certain kinds of wrongful acts, paradigmatically serious crimes, morally deserve to suffer a proportionate punishment;
2. That it is intrinsically morally good—good without reference to any other goods that might arise—if some legitimate punisher gives them the punishment they deserve; and
3. That it is morally impermissible intentionally to punish the innocent or to inflict disproportionately large punishments on wrongdoers.”^35^

The problems with this view are sociological on the one hand, and philosophical on the other. On the sociological side, the problem is that despite the constraints implied by retributivism’s third claim—the prohibition on disproportionate punishment—retributivist ideas often seem to be invoked to justify harsh criminal-justice systems, which by many critics’ accounts are not only inhumane but even counterproductive. While incarcerating many people, often in tough conditions, these systems may still fail to deter crime and even contribute to vicious circles of more crime and violence in the societies in question.^36^ Retributivism is arguably the doctrine behind much of the contemporary approach to criminal justice in the United States, which has been widely criticized for being neither sufficiently humane nor sufficiently effective.^37^ As observed by Francis Allen in the early 1980s, the “rehabilitative ideal”, which had long been a guiding principle in criminal punishment, has been in decline in the United States since the 1970s.^38^ Similar trends can be seen elsewhere too. As David Garland comments in his 2001 book,

“[f]or most of the twentieth century, penalties that appeared explicitly retributive or deliberately harsh were criticized as anachronisms that had no place within a ‘modern’ penal system. In the last twenty years, however, we have seen the reappearance of ‘just deserts’ retribution as a generalized policy goal in the US and the UK, initially prompted by the perceived unfairness of individualized sentencing. This development has certainly promoted the concern for proportionality and fixed sentencing for which its liberal proponents had hoped. But it has also re-established the legitimacy of an explicitly retributive discourse, which, in turn, has made it easier for politicians and legislatures to openly express punitive sentiments and to enact more draconian laws.”^39^

I must emphasize that the retributivist view consisting of claims (1)–(3) above does not *logically* entail a commitment to a harsh—let alone draconian—approach to punishment. Indeed, as a philosophical view, it is compatible with the assumption that only light punishments are ever deserved, and some philosophical proponents of retributivism have explicitly criticized overpunishment.^40^ In theory, retributivism is primarily a view about the point, purpose, and justification of punishment, not a view about its severity. Yet, once we consider public discourse instead of academic philosophy, the worry is that an appeal to retributivist ideas may contribute to a punitive culture in the societies in question.

However, even if we thought that retributivist discourse need not have any bad societal effects, retributivism still has a philosophical problem. There seems to be no good reason for accepting retributivism’s claim that wrongdoers *deserve* to suffer a punishment that takes the form of a setback to their interests and that inflicting this setback upon them is *intrinsically good*, irrespective of any other goods the punishment may produce.^41^ According to a widely accepted moral principle,

intentionally inflicting a harm upon someone—intentionally setting back their interests—is justifiable *only if* this is *strictly necessary* for something that takes sufficiently strong moral priority, and thus if it is, in a sense, unavoidable.

I find it difficult to square retributivism with this principle. First of all, it seems implausible that the harm that is intentionally inflicted upon a wrongdoer in a system of retributive justice is always necessary in the required sense. Retribution is not necessary, for instance, if there are alternative ways of achieving deterrence and/or compensation or if the retributive punishment could be replaced by a more constructive process, such as an acknowledgement of the wrong that was committed, combined with a requirement to compensate the victim(s) of that wrong and somehow to make up for it and/or to serve the community. Moreover, even in cases in which inflicting a harm on someone is genuinely necessary and unavoidable, such as in certain cases of self-defence, this is always regrettable and not something we would wish to describe as “intrinsically good”. So, even if wrongdoers genuinely deserved to be punished, the claim that inflicting this punishment upon them is *intrinsically good*—retributivism’s second claim—would still seem hard to defend.

It is certainly plausible that if someone has committed a wrong for which they are responsible, they are under an obligation to apologize, to put things right, and to engage in certain compensatory and/or restorative activities. Deriving such an obligation from the wrongdoing seems straightforward and non-mysterious—and certainly not in breach of any familiar moral principles. It is also plausible that someone responsible for a serious wrong may have forfeited the right not to be interfered with for protective purposes, possibly in the form of imprisonment, in case they continue to pose a significant risk to the community. The interference will then be *instrumentally justified* by reference to its practical necessity for protecting the rights of others.

By contrast, it is much more of a stretch to derive the *intrinsic goodness* of inflicting a retributive harm on someone from the fact that they have committed a wrong. If I wrong you, then I certainly incur an obligation to apologize to you and to put things right, as far as possible. But why should it suddenly become *intrinsically good* for some third party, such as the state, to inflict some harm on me as a punishment, irrespective of any other goods that are promoted by this, such as deterrence, protection of the community, compensation, or education?

My sense is that while retributivism might capture some people’s pre-theoretic intuitions about punishment, some of its core claims, namely (1) and (2) in Walen’s characterization of the view, lack any compelling philosophical grounding. Only claim (3) is a welcome prohibition on unjustified and disproportionate punishment. Apart from the fact that claims (1), (2), and (3) might jointly capture certain pre-theoretic intuitions, I find it hard to see why we should independently believe claims (1) and (2).

Since a retributivist account of punishment suffers from these philosophical difficulties, quite apart from any political reservations we may have about retributivist discourse, we have good reasons to look for a non-retributivist alternative. There are several competing accounts of punishment on offer, and this paper is not the place to settle the question of which account is best. Broadly speaking, there are those proposals which, despite giving up the idea of retribution, continue to treat responsibility attributions as central to a system of criminal justice and those that largely dispense with responsibility attributions.

Some of the proposals made by responsibility sceptics such as Gregg Caruso and Derk Pereboom fall into the latter camp.^42^ Both Caruso and Pereboom propose to reconceptualize the idea of criminal justice around an analogy with public health. Just as, in many countries, COVID-19 patients are required to quarantine until they are sufficiently recovered to generate no further infection risks for others, so criminals who have committed serious offences may be required to “quarantine” for some time to protect the community or to give them a chance to undergo rehabilitation. Caruso suggests that public-health ethics provides a useful and humane framework for justifying criminal sanctions on the model of quarantine in the domain of public health, without invoking any logic of retribution.

I can see the appeal of such a framework, especially from a consequentialist perspective, and I agree that if we were forced to give up the idea of responsibility altogether—perhaps in response to the challenges from neuroscience—the public-health analogy would offer a promising way to reconceptualize criminal justice.

That said, my own realism about free will and responsibility leads me to prefer a more deontological approach, in which responsibility attributions continue to play an important role in criminal justice. The approach I favour is a restorative-justice one in which offenders are still recognized as responsible for the wrongs they have committed and in which they incur the obligation not only to accept accountability but also to restore moral relations, for instance by apologizing to the victims of those wrongs, by offering compensation and, where possible, making up for those wrongs, and/or by engaging in appropriate forms of community service.^43^ Crucially, the process is a constructive and forward-looking one. The aim is not to set back the interests of the offender but to restore communal relations within the relevant moral community. And even if, as a byproduct of the process, the offender’s interests are set back, which may sometimes be unavoidable, this is not the point and purpose of the punishment.^44^

While this is clearly a non-retributivist approach to punishment, it is one that still relies on realism about free will and responsibility. One important step towards achieving restorative justice is precisely the acknowledgement that a wrongdoer is responsible for something he or she has done, that he or she ought to have acted otherwise, and that he or she is now under an obligation to put things right. Regardless of the material burden that “putting things right” ends up imposing on the offender (the burden could be modest, or it could be significant, depending on the wrong in question), the very acknowledgement that the offender is responsible is already a key element in the restoration of good relations within the relevant moral community.

In sum, I agree with Michael Moore that something would be lost from the perspective of criminal justice if we had to give up the ideas of free will and responsibility, and that it is important to defend those ideas against the challenges from neuroscience. But I also agree with sceptics such as Gregg Caruso and Derk Pereboom that a retributivist approach to punishment is unattractive and that we should reform our criminal-justice systems in a non-retributivist manner. The reason I reach this conclusion, however, is not that I think that neuroscience has refuted free will, but that there are good independent reasons for giving up retributivism and for replacing it with an approach to criminal justice that takes the idea of responsibility seriously while reconceptualizing punishment in a constructive and forward-looking way.

## Footnotes

1 These quotes come from Eagleman (2015, pp. 78–79 and pp. 104–105), quoted in Moore (2020).

2 Ibid., p. 177, also quoted in Moore (2020).

3 See Moore (2020).

4 See Moore (2020, abstract).

5 For a full defence of compatibilist libertarianism, see List (2019).

6 See Moore (2020, ch. 1).

7 See List (2019).

8 See Moore (2020, ch. 8).

9 See Moore (2020, p. 268).

10 My own wording of the second thesis, especially the reference to “settling”, is inspired by Helen Steward’s exposition of libertarianism, in Steward (2012).

11 See, e.g., Kane (1996).

12 See, e.g., O’Connor (1995).

13 See Moore (1912).

14 See ibid., p. 12.

15 This definition is based on Fara (2008). For critical discussions of the dispositional analysis of the ability to do otherwise, see, e.g., Clarke (2009) and Whittle (2010).

16 See Moore (2020, ch. 8).

17 See Caruso (2020), emphasis original.

18 See Lewis (1973) and Robert (1968).

19 Compatibilists in the tradition of G.E. Moore are likely to disagree with me here and argue that the compatibility of the relevant conditionals with the impossibility of doing otherwise is a feature of their account, not a bug. I am presenting what I regard as a commonsense objection to this form of compatibilism. I recognize that the objection relies on incompatibilist intuitions (which, however, may be pre-theoretically widely shared).

20 For critiques of the ascription of basic desert moral responsibility, see, e.g., Caruso (2020) and Pereboom (2001).

21 See Hurley (2003, p. 16).

22 I here draw on my book (List 2019).

23 My argument here is based on List (2014, 2019). The present exposition of the indispensability argument is most similar to the one in List (2022).

24 See Quine (1977) and Fine (1984).

25 See List (2019). My exposition here further draws on List (2022).

26 See Dennett (1987).

27 I have previously made this point, for instance, in my response to critics in List (2020).

28 My argument here is based on List (2014).

29 See List (2014) and List and Pivato (2015).

30 Diagram 2 is reproduced from List (2019).

31 See Butterfield (2012). Related points can be found in Yoshimi (2012) and, with a slightly different interpretation (namely, observational indistinguishability of deterministic and indeterministic descriptions), Werndl (2009). For my own earlier analysis of the level-relativity of the determinism-indeterminism distinction, see, in particular, List and Pivato (2015), where we also respond to some objections.

32 See Moore (2020, pp. 448–449 and pp. 277–278, respectively). The relevant references on levels are Bechtel (2008) and Craver (2007).

33 See Moore (2020, p. 278).

34 Indeed, some leading critics, such as Caruso (2021), offer several arguments against retributivism, only some (but not all) of which concern the issue of free will.

35 See Walen (2021). I note, however, that not all retributivists accept all three claims in precisely this form. Berman (2022), for instance, defends retributivism against some influential objections and describes a retributivist theory whose primary claims are that “culpable wrongdoers deserve to experience their wrongdoing as personally costly to them, and the state assumes a pro tanto duty to cause wrongdoers to experience those costs in virtue of barring retaliation by victims and their surrogates” (p. 1).

36 See, e.g., Caruso (2021).

37 On the shift in emphasis from rehabilitation to retribution in American criminal justice, see, e.g., Allen (1981) and Garland (2001). Note that, according to World Prison Brief data (accessed on 19 October 2022), the US has one of the highest incarceration rates in the world (at 505 per 100,000 people, compared to Germany’s 67 and Norway’s 57). Furthermore, prison conditions in the United States are often considered harsher than in other countries at similar levels of economic development.

38 See Allen (1981).

39 See Garland (2001, pp. 8–9).

40 See, e.g., Husak (2020). Husak suggests that “a retributive penal philosophy should not be blamed for contributing to our present epidemic of mass incarceration and tendency to over-punish” (abstract), and he argues that, by retributivist standards, “many persons deserve less punishment than our legal system currently inflicts” (p. 3). In this context, recall again retributivism’s claim (3), the prohibition on disproportionate punishment.

41 For an in-depth philosophical critique of retributivism, see, e.g., Shafer-Landau (1996). I can here give only a brief sketch of my philosophical reservations about retributivism.

42 See Caruso (2021) and Pereboom (2001).

43 For discussion, see also Hoskins and Duff (2021).

44 Despite some of the differences between my views about criminal justice and the views of Caruso and Pereboom, there is still some common ground between us. I refer readers to the forward-looking account of moral responsibility that Caruso and Pereboom present in their 2022 book.
